# Current considerations for heart-kidney transplantation

**DOI:** 10.3389/frtra.2022.1022780

**Published:** 2022-10-26

**Authors:** Syed Adeel Ahsan, Lamees I. El Nihum, Priya Arunachalam, Nina Manian, Qasim Al Abri, Ashrith Guha

**Affiliations:** ^1^DeBakey Heart and Vascular Center, Houston Methodist Hospital, Houston, TX, United States; ^2^Texas A&M College of Medicine, Bryan, TX, United States

**Keywords:** heart-kidney transplantation, heart transplant (HTx), kidney transplant, heart failure, cardiorenal syndrome (CRS), orthotopic heart transplant (OHT), deceased donor kidney transplant

## Abstract

Cardiorenal syndrome is a complex syndrome characterized by dysfunction of the heart and kidneys in an interdependent fashion and is further divided into different subtypes based on primary organ dysfunction. Simultaneous Heart-Kidney transplantation is the treatment of choice for end-stage irreversible dysfunction of both organs, however it may be avoided with determination of cardiorenal subtype and management of primary organ dysfunction. This article discusses types of cardiorenal syndrome, indications and concerns regarding the use of simultaneous heart-kidney transplantation, and outlines algorithms for determination of need for dual vs. single organ transplantation.

## Introduction

Concurrent dysfunction of the heart and kidneys, referred to as cardiorenal syndrome (CRS), is a well-described phenomenon, occurring due to the significant interactions of these organs in physiological and pathological states (1). This complex relationship persists even after definitive therapy of either organ with transplantation. While kidney transplantation is the treatment of choice for end-stage renal disease (ESRD), cardiovascular disease is a major cause of post-transplant morbidity and mortality, and represents the leading cause of death in kidney transplant patients with a functioning graft (2, 3). Conversely, renal dysfunction is a significant contributor to long-term mortality post heart transplantation. Therefore, simultaneous heart-kidney (sHK) transplantation has evolved as a recognized therapy for advanced heart failure with advanced chronic kidney disease (CKD) (4). As a caveat to multi-organ transplantation, solitary organ transplantation in both heart failure and advanced CKD is associated with decreased adverse events related to secondary organ dysfunction, suggesting that multiorgan transplantation is not always necessary (5, 6). When patients present with simultaneous organ dysfunction, it is imperative to determine the degree of reversibility of secondary organ dysfunction in order to make a decision regarding definitive therapy with transplantation.

This poses the question: for patients referred for simultaneous heart-kidney transplantation, can the patient benefit from only one of the organs being transplanted?

## Cardiorenal syndrome and sHK transplantation

Of multiorgan transplants, sHK transplants have experienced the largest increase in frequency from 2015 to 2020 (7). This has occurred even though debate exists regarding the exact population of patients that would benefit from dual organ transplantation, and there is notable variability in kidney function of sHK recipients by center, with median estimated glomerular filtration rate (eGFR) ranging from 19 to 59 ml/min/1.73 m^2^ from 2014 to 2019 (8). This debate, and resulting variability, is driven by the inability to predict the potential for renal recovery in patients with heart failure or recovery of heart function in patients with renal failure.

While it is established that heart transplant alone is contraindicated in patients with heart failure requiring dialysis, sHK transplant in patients without sufficiently deranged renal function shows no benefit over heart transplant alone (5, 9, 10). Additionally, sHK transplant has the potential for worsened kidney graft survival as compared to kidney transplant alone due to alterations in hemodynamics, use of vasopressors, and use of cardiopulmonary bypass (11). Conversely, sHK transplant is associated with improved cardiac graft survival, driven by lower rates of cardiac allograft rejection events and coronary allograft vasculopathy (9, 12, 13). Therefore, the indications for sHK transplant in patients with heart failure have varied over 30 years of conferences and societal meetings. This culminated in a recent consensus conference recommending sHK transplant in patients with eGFR <30 ml/min/1.73 m^2^, and allowing for consideration of sHK transplant in patients with eGFR 30–44 ml/min/1.73 m^2^ with evidence of intrinsic CKD (14–19).

In patients with advanced CKD, namely eGFR <30 ml/min/1.73 m^2^, or ESRD, the presence of hemodynamic volume overload, arterial remodeling, arterio-venous shunts, and uremia contribute to the development and worsening of cardiorenal syndrome. In these patients, kidney transplant has the potential to improve cardiac function through reverse cardiac remodeling and decreased volume overload. A single-center study at Cleveland Clinic from 2003 to 2013 investigated patients with left ventricular (LV) dysfunction who underwent kidney transplant alone; comparison of baseline and post-transplant echocardiograms demonstrated an improvement in LV ejection fraction with significant improvement in LV diastolic function, LV mass, and right ventricular systolic pressure (6).

Decision making regarding solitary as compared to dual organ transplant will predominantly depend on the primary etiology of organ dysfunction. There are five described subtypes of cardiorenal syndrome that encompass the spectrum of acute or chronic dysfunction of either the heart or kidneys because of acute or chronic dysfunction of the other organ. Type 1 is an acute cardiorenal syndrome due to heart failure and cardiogenic shock in which the acute heart failure and resulting congestion causes an acute kidney injury (AKI). Type 2 is a chronic cardiorenal syndrome where chronic heart failure leads to CKD. Type 3 is described as acute renocardiac syndrome, where AKI leads to volume overload, an inflammatory surge, and metabolic disturbances secondary to uremia, culminating in heart failure. Type 4 is chronic renocardiac syndrome, in which left ventricular hypertrophy and chronic heart failure result from CKD-associated cardiomyopathy. Finally, type 5 is secondary cardiorenal syndrome due to a systemic process such as amyloidosis, sepsis, or cirrhosis, resulting in simultaneous heart and kidney failure (20).

Underlying the concerns for the benefit of multiorgan transplant are the ethical considerations regarding potential harm in removing organs from the donation pool. These considerations revolve around beneficence, what benefits the individual patient, and utility, what benefits the greatest number of patients. In the application of these ethical principles to sHK transplant, the benefit of replacing both organs will depend on the degree of secondary renal or cardiac dysfunction. Some allocation procedures for sHK transplant may assign organs to individuals who receive less benefit in terms of life-years gained; additionally, patients who receive kidneys through the multiorgan transplant system generally receive higher quality kidneys as measured by the kidney donor profile index (KDPI), undermining the current matching scheme for kidney transplant alone (21). The Organ Procurement and Transplantation Network/United Network of Organ Sharing published a Liver-Kidney Transplant policy in 2017 to combat kidney over-utilization, which incorporated a “safety net,” wherein patients with renal failure in the first year post-liver transplant would be prioritized for kidney transplant (22). Adoption of a similar policy for patients being considered for sHK has been proposed, and was shown to have favorable cost-effectiveness, particularly in patients with moderate or high likelihood of kidney dysfunction reversibility (19, 23, 24). However, these proposals have not yet been codified into policy.

A sHK transplant is beneficial to patients who cannot recover function in either organ with single-organ transplantation. However, because of the interdependence of the heart and kidney, there is potential for patients to benefit from transplantation of one organ alone. We discuss the following algorithms to determine if replacement of one organ is sufficient instead of a simultaneous heart-kidney transplant. Separate algorithms for kidney failure in heart transplant candidates and heart failure in patients with established kidney dysfunction were used in two different cases.

## Case 1: Heart transplant alone to reverse kidney failure

### Case history

A 62-year-old woman with burnt out hypertrophic cardiomyopathy (HCM) with a history of LV aneurysmectomy, mitral valve replacement, and ventricular tachycardia was referred for consideration for heart-kidney transplant. The patient had New York Heart Association (NYHA) class IIIB-IV symptoms, with dizziness and frequent falls at home. On exam, her blood pressure was 90/50 mmHg and her heart rate was paced at 60 beats per minute. She appeared frail, with jugular venous distension and cool extremities. Her baseline creatinine was 1.68 mg/dL and eGFR ranged between 25 and 40 ml/min/1.73 m^2^. Urinalysis was negative for proteinuria and casts. Her echocardiogram revealed a small LV with an apical patch, a bioprosthetic mitral valve, decreased right ventricular (RV) function, and no significant valvular dysfunction. Renal ultrasound demonstrated normal sized kidneys with mild renal disease. Her initial hemodynamics at a heart rate of 60 and blood pressure of 92/52 mmHg had a right atrial (RA) mean pressure of 14 mmHg, an RV pressure of 44/4 mmHg, a pulmonary arterial (PA) pressure of 44/28 (mean 33) mmHg, and a mean pulmonary capillary wedge pressure (PCWP) of 28 mmHg with a cardiac output of 2.4 L/min and cardiac index of 1.6 L/min/m^2^ by thermodilution.

Given hemodynamics consistent with cardiogenic shock the patient was supported with intra-aortic balloon pump placement and milrinone infusion. During this period she had repeated eGFR measurements between 40 and 60 ml/min/1.73 m^2^. After multi-disciplinary discussion with the renal transplant team and heart failure cardiologists, the patient was listed for and underwent heart transplant alone. Her post-operative creatinine was 1.0 mg/dL and her eGFR increased to over 60 ml/min/1.73 m^2^, demonstrating a significant improvement in her kidney function.

### Discussion

Cardiac dysfunction, whether acute or chronic, can impact renal function in multiple ways. The neurohormonal dysregulation of chronic heart failure and activation of the renin-angiotensin aldosterone system (RAAS) plays a significant role in kidney injury and progression of renal disease. The consequences of increased renin, angiotensin II and aldosterone include increased sodium reabsorption, as well as production of pro-inflammatory and pro-fibrotic peptides, which result in worsening renal function along with injury and hemodynamic stress to the heart. Hemodynamic stress may include low cardiac output and central venous congestion. Low cardiac output states result in inadequate renal blood flow, which increases RAAS activation, thereby worsening renal afferent arteriolar vasoconstriction, and leads to oxidative stress and hypoperfusion injury to the kidneys. Central venous congestion, represented by elevated central venous pressure (CVP), results in elevated intraabdominal pressure, decreased renal capillary pressure gradient, reduced renal blood flow and glomerular filtration rate, ultimately contributing to decreased kidney function (1, 25, 26).

**Figure 1:**
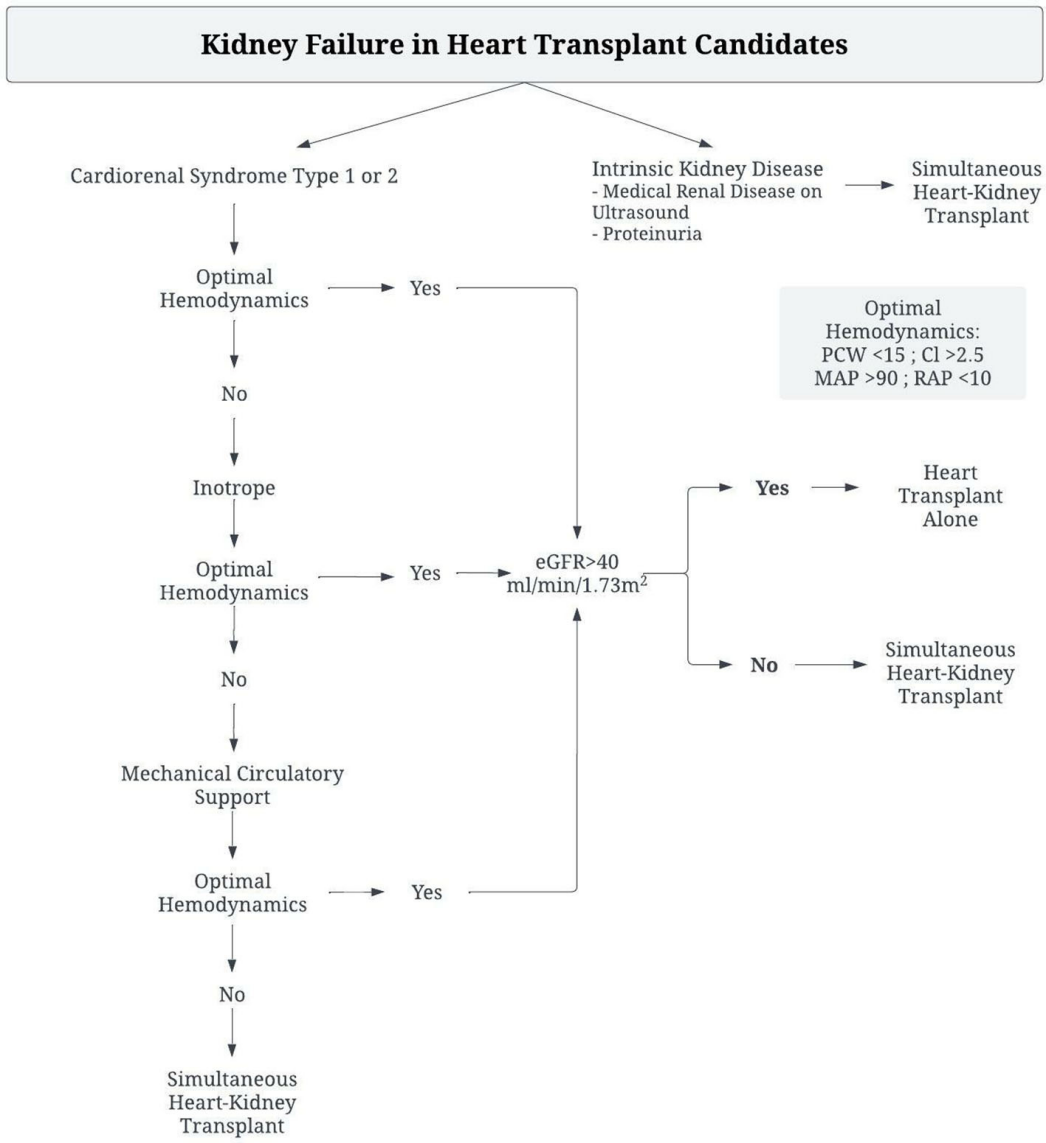
Algorithm for management of Heart-Transplant candidates with renal dysfunction to determine need for Simultaneous Heart-Kidney Transplant.

Management of kidney failure in heart transplant candidates should therefore be tailored toward identification of the disease process, and interventions to remedy the resultant pathophysiologic derangements, as reflected in Figure 1. Patients with clear evidence of intrinsic renal disease, such as medical renal disease on ultrasound or significant proteinuria, likely have irreversible renal dysfunction and require consideration of sHK transplant. However, in the absence of such findings, invasive hemodynamic assessment is necessary as sHK transplant should be considered if renal function is impaired despite optimal hemodynamics. Hemodynamic derangements in these patients can be broadly classified into 3 states. Patients with a low cardiac index but normal filling pressures are primarily suffering from a hypoperfusive state, and improvement in perfusion by inotropes or mechanical circulatory support devices is necessary to determine renal recovery. Those with a low cardiac index, elevated PCWP, but normal CVP are similarly suffering from a low perfusion state and require therapy to improve cardiac output. In patients with a low cardiac index, elevated PCWP and elevated CVP the impairment is related to a decreased gradient across the renal capillaries, thus these patients require both support to increase the cardiac output as well as therapies to reduce central venous congestion.

This patient's primary disease process was CRS type 2, with a low cardiac output and elevated filling pressures. Given her lack of structural abnormality, the decision was made to optimize hemodynamics with mechanical circulatory support and inotrope therapy. As a result, she had improvement in her markers of renal function, and was able to tolerate heart transplant alone with excellent post-operative renal function. She was spared unnecessary dual organ-transplant, and therefore did not utilize a donor organ that would have been of limited benefit to her.

## Case 2: Kidney transplant alone to reverse heart failure

### Case history

A 52-year-old male patient with ESRD for many years had been listed for kidney transplant for 6 years. The patient had a newly recognized drop in LV ejection fraction (LVEF) and two admissions for volume overload in the last 6 months. He had NYHA class III symptoms with dyspnea on exertion, and his persistent hypotension did not allow for up-titration of goal-directed medical therapy for heart failure. He was therefore referred for simultaneous heart-kidney transplant. On physical exam, his blood pressure was 95/50 mmHg and heart rate was 85 beats per minute. A prominent S3 was appreciable, and the patient had bilateral lower extremity edema. Echocardiogram revealed a dilated LV, mild mitral regurgitation, mildly depressed right ventricular function, severely depressed LV function with LVEF of 25–30%, and pulmonary arterial systolic pressure of 60–65 mmHg. Cardiac MRI did not show evidence of infiltrative disease. Coronary angiogram revealed no left main or left anterior descending (LAD) disease, 70% occlusion in a first diagonal branch of the LAD, and 30–40% stenosis in the left circumflex and right coronary arteries. An arteriovenous (AV) fistula study showed brachial artery flow of 2,700 mL/min. Initial right heart catheterization at a heart rate of 60 and blood pressure of 95/50 mmHg had an RA mean pressure of 18 mmHg, RV pressure of 60/4 mmHg, PA pressure of 60/28 (mean 38) mmHg, and a mean PCWP of 28 mmHg with a cardiac output of 7.3 l/min and cardiac index of 3.6 l/min/m^2^ by thermodilution.

Given these findings, he was diagnosed with high-output heart failure related to increased flow through the AV fistula. The patient underwent fistula revision, resulting in a decrease in brachial arterial flow to 1,100 mL/min. A repeat transthoracic echocardiogram 6 months from the intervention revealed a normal LV size, improvement in LV function to normal with LVEF 60–65%, trace mitral regurgitation and normal RV function. Repeat right heart catheterization at a heart rate of 60 and blood pressure of 95/50 mmHg revealed an RA mean pressure of 5 mmHg, RV pressure of 41/2 mmHg, PA pressure of 41/16 (mean 24) mmHg, and PCWP of 16 mmHg with a cardiac output of 6.0 l/min and cardiac index of 3.1 l/min/m^2^ by thermodilution.

Given the patient's sustained improvement in left ventricular parameters, he was listed for kidney transplant alone.

### Discussion

Renal dysfunction leading to cardiac injury and dysfunction occurs throughout the spectrum of progressive renal disease. Acute kidney injury is difficult to identify as a causative agent in cardiac dysfunction, as in the case of CRS 3, due to the array of comorbid conditions contributing to AKI. AKI is associated with a dramatically increased risk of cardiovascular mortality and major cardiovascular events, whether by fluid overload leading to pulmonary edema, or electrolyte abnormalities leading to arrythmias (20, 27). Chronic kidney disease in the early stages can lead to development of comorbid conditions such as hypertension, anemia, and derangements in nutritional status, all of which are risk factors for cardiac disease. The chronic inflammatory state induced by renal injury results in the generation of pro-inflammatory biomarkers that are implicated in the development of both heart failure and coronary atherosclerosis. Progressive disease leads to abnormalities of calcium and phosphate, accumulation of uremic toxins, and further chronic inflammation. Eventually, ESRD and fluid shifts resulting from dialysis, worsening electrolyte and calcium/phosphate homeostasis, as well as worsening anemia and malnutrition all result in progressive myocyte dysfunction, cardiac remodeling and fibrosis, atherosclerotic disease and arrythmias (1, 20, 26). In certain cases, high output heart failure can result from AV fistulas surgically created for dialysis. The acute decrease in systemic vascular resistance following fistula creation causes an increase in sympathetic nervous system activity leading to increased contractility and heart rate. Over time, blood volume increases causing elevated venous return and cardiac filling pressures (28, 29). High flow fistulas, particularly those with flow volume >2 L/min, are associated with the development of worsening RV dysfunction, LV hypertrophy, pulmonary hypertension, and eventually heart failure (30–32).

**Figure 2:**
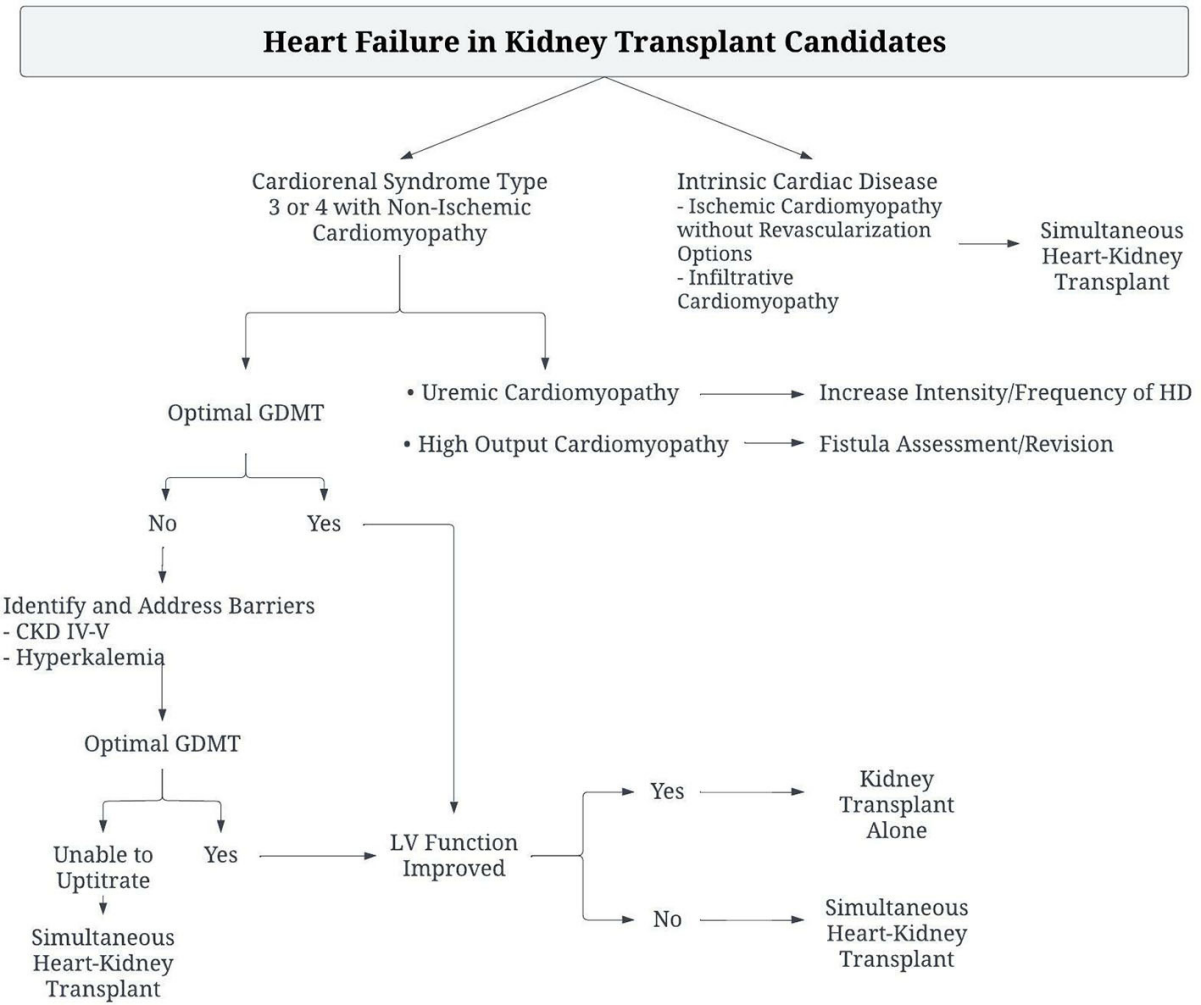
Algorithm for the management of Kidney transplant candidates with heart failure to determine need for Simultaneous Heart-Kidney Transplant.

Management of patients with ESRD presenting with heart failure should involve identification of treatable causes of heart failure, such as ischemic heart disease or infiltrative disease. Patients with non-ischemic cardiomyopathy should be treated with guideline directed medical therapy for heart failure, along with management of volume status and anemia. Patients should be evaluated for high-ouput heart failure and treated if necessary, as correction of the high output state has been associated with improvement in LV mass, biventricular volumes and NT-proBNP levels (33). Unused AV fistula sites should be ligated, and flow through other sites measured. Reduction in AV fistula flow may be attempted with precision banding, or ligation and grafting (34, 35). If heart failure persists, AV fistula closure should be considered. If the heart failure disease state and myocardial dysfunction are unable to be corrected by these methods, the patient should be considered for sHK transplant (Figure 2).

## Conclusion

Patients with concomitant kidney and heart dysfunction are difficult to manage; as a result, many patients are referred for simultaneous heart-kidney transplant. However, algorithm-based management of these patients may allow for correction of secondary organ dysfunction, and thereby allow for single organ transplant alone. In patients with heart failure, optimization of hemodynamics with appropriate support can allow for heart transplantation alone and reversal of cardiorenal syndrome due to the benefits of improvement in renal congestion and perfusion. Similarly, GDMT, optimal dialysis, and correction of high output states can reverse cardiomyopathy in patients with CKD, allowing for kidney transplantation alone and conferring the benefits of optimized kidney function for heart function.

## Ethics statement

The authors attest they are in compliance with human studies committees and animal welfare regulations of the authors' institutions and Food and Drug Administration guidelines, including patient consent where appropriate.

## Author contributions

SA provided final drafts, figures, and discussion contents as well as references. LE and PA provided initial drafts, as well as content, and reviewed further drafts. NM and QA reviewed and assisted with drafts of the manuscript. AG set out the outline of the paper, as well as figure concept and design, and provided final review. All authors contributed to manuscript revision, read, and approved the submitted version.

## Conflict of interest

The authors declare that the research was conducted in the absence of any commercial or financial relationships that could be construed as a potential conflict of interest.

## Publisher's note

All claims expressed in this article are solely those of the authors and do not necessarily represent those of their affiliated organizations, or those of the publisher, the editors and the reviewers. Any product that may be evaluated in this article, or claim that may be made by its manufacturer, is not guaranteed or endorsed by the publisher.

## Abbreviations

sHK: simultaneous heart-kidney.
